# Awareness and Attitudes about Disease Mongering among Medical and Pharmaceutical Students

**DOI:** 10.1371/journal.pmed.0030213

**Published:** 2006-04-11

**Authors:** C. Jairaj Kumar, Abhizith Deoker, Ashwini Kumar, Arunachalam Kumar, B. M Hegde

**Affiliations:** **1**Kasturba Medical College MangaloreKarnatakaIndia; **2**Manipal Academy of Higher EducationManipal, KarnatakaIndia


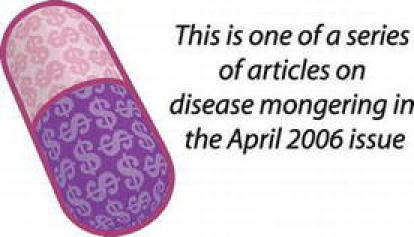


Pharmaceutical companies throughout the world market their products aggressively through a variety of promotional campaigns [ 1]. In India, these marketing practices pose a greater problem because the restrictions on drug dispensing are very limited—drugs often being dispensed without a prescription from a licensed physician. The companies take full advantage of this situation. As many patients in India are poor and illiterate, and lack information on health care, they often visit local pharmacists or quacks for medical advice. Pharmacists routinely dispense drugs illegally over the counter. We visited 40 local pharmacy stores for medical advice for a feigned medical ailment, and we found that all 40 pharmacists dispensed drugs, including expensive antibiotics [ 2].

Pharmaceutical promotional campaigns in India, unlike those in developed countries (where pharmacists have little influence on drug sales), are not only aimed at changing the prescribing habits of physicians but also at pharmacists and quacks. Pharmaceutical companies in India offer various schemes and incentives (including television sets, motorcycles, and the opportunity for higher profit margins) to lure pharmacists into buying more drugs than they would normally need. As a result, the pharmacists make every effort to sell these drugs to patients visiting them for medical advice. They may also associate themselves with quacks or physicians in their efforts to shift their stock of the drugs.

In developed countries, dubious pharmaceutical marketing practices would soon attract the attention of watchdog bodies and social activists, but in India they go undetected. We believe that this situation demands proactive action on the part of the medical profession and also of the government.

The efforts of the pharmaceutical industry to medicalize human life should be resisted. We do not wish India to be in the same position as the countries of the West, where adverse drug reactions are responsible for a significant proportion of hospital admissions and require millions of outpatient visits and corrective measures. In the United States, for example, there are about 100,000 deaths due to medical errors every year, of which about 7,000 are attributed to drug reactions [ 3].

We believe it is important to assess current awareness about disease mongering among medical and pharmaceutical students, as pharmaceutical promotional campaigns are aimed at both professions. Assessing current awareness could provide a basis for further research, leading to the development of effective measures that will raise awareness levels and motivate students to participate in future campaigns that seek to combat disease mongering.

Most medical and pharmaceutical students in India are not aware of the issue of disease mongering; neither do most of them know that recent audits have shown medical interventions and adverse drug reactions to be major causes of death and disability in the US [ 4].

Articles have been published warning the profession about disease mongering [ 5–7], but for the most part these warnings have not been heeded. One is reminded of Aristotle, who so rightly observed that “truth could influence only half a score of men in a century, while falsehood and mystery would drag millions by the nose.”

We prepared a 20-item questionnaire (Text S1) about disease mongering and the influence of the drug industry on clinical practice. The questionnaires were distributed among a random sample of 250 final-year medical and 250 final-year pharmaceutical students. The overall response rate was 406 out of 500 (81.2%), comprising 199 medical and 207 pharmaceutical students. Of the medical students, 30 out of 199 (15%) were able to explain disease mongering with relevant examples. Of the pharmaceutical students, 114 out of 207 (55%) were able to do so, suggesting that awareness of the problem was much greater among these students. Interestingly, however, 87 out of 114 pharmaceutical students believed the government, not the pharmaceutical industry, was responsible for the problem.

All the students, both medical and pharmaceutical, said they had frequently seen drugs dispensed without prescription. They had also often seen patients visit local pharmacists for medical advice. They agreed that both practices were unethical. However, both the medical and the pharmaceutical students were unaware of the incentives offered by drug companies to pharmacists for buying their drugs, which lead to unethical dispensing.

We believe that our small project, despite its inherent limitations, has thrown some light on the situation. Pharmaceutical students, who are exposed to the drug industry to some extent during their studies, have some idea of the magnitude of the problem, while the majority of medical students have no idea that even their textbooks are written with the help of money that comes from drug companies [ 8]. We need to make a more concerted attempt to educate the student community of all the health-care professions, in order to counter this unfair tendency. The government should undertake major initiatives to ensure that drugs are only dispensed with a prescription from a licensed physician. Medical associations and medical college administrators should alert their members to cross-check the information provided in drug company literature. Medical students should be warned about disease mongering through the display of posters, and through the organization of essay competitions and interactive plays. Students can play a further role by conducting regional and national surveys of the awareness of the public concerning this serious issue.

## Supporting Information

Text S1 20-Item Questionnaire about Disease Mongering and the Influence of the Drug Industry on Clinical Practice (25 KB DOC).
