# Supplementary material for: Awareness and Attitudes about Disease Mongering among Medical and Pharmaceutical Students
Source: PLoS Med. 2006 Apr 11;3(4):e213. doi: 10.1371/journal.pmed.0030213 (PMC1434511; doi:10.1371/journal.pmed.0030213)
Supplement: Supplementary file 1 [file pmed.0030213.sd001.doc]

Dear friends,

Please take some time filling the proforma…….

PROFORMA

1. Do you know about any pharmaceutical marketing strategies aimed at encouraging self- medication among patients by creating fear or changing prescribing habits of physicians? (It is now termed as Disease mongering or selling of sickness)

Yes	No

2. If yes what according to you is disease mongering.

………………………………………………………………………………………………………………………………………………………………………………………………

3. If yes, quote some examples of disease mongering.

………………………………………………………………………………………………………………………………………………………………………………………………………………………………………………………………………………………………

4. If yes, where you got information from?

a) News paper /T.V b) Internet c) scientific journals d) Teachers e) books f) any others

5. Who do you think is mainly responsible for disease mongering?

a) Doctor b) Pharmaceutical industry c) people d) Government e) any others specify

7. According to you is it ethical to take complements from pharmaceutical industries by Doctor?

a) Yes	b) NO

8. Have you noticed any medical conferences without pharmaceutical sponsorers?

a) Yes	b) No

If yes specify……………………………………………………………………………

9. Why do you think pharmaceutical industry sponsors medical conferences?

……………………………………………………………………………………….

10. How often have you seen drugs being dispensed by pharmacists without a prescription from a medically qualified doctor?

11. Do you think dispensing drugs without a prescription from physician is unethical?

a) Yes	b) No

12. If yes why do you think it is unethical?

a) Medical reasons causing side effects	b) Unethical means of marketing c) a&b

If you feel it is both a&b can you substantiate your answer with examples how it can lead to unethical means of marketing in large scale……………………………………

13. Do you any idea regarding the amount of money a pharmaceutical industry invests in developing a new drug?..............................................

14. How do you rate about the influence of pharmaceutical companies on medical practice?

a) Very high b) high c) moderate d) very less e) don’t know

15. Are you ready to participate in any campaign to attenuate of selling of sickness/disease mongering?

a) Yes	b) No

16. Who according to you should take initiative in preventing disease mongering?

a) Govt of India	b) medical Organizations (IMA/MCI) c) student organizations

d) Don’t know

17. Have you heard of any journal/organizations taking initiative in preventing disease mongering?

If yes specify………………………………………………………………………….......

18. Have you heard of any books written on disease mongering?

If yes please name it…………………………………

19. Do you think making students aware during their academic course can attenuate disease mongering?

a) Yes	b) No

20. Do you feel that by participating in campaign against disease mongering will hamper your practice in future/

a) Yes	b) No

**THANK YOU…….**
